# Exploring Food Choice Influences in Athletes and Active Populations in Ireland: A Cross-Sectional Study

**DOI:** 10.1016/j.cdnut.2025.104568

**Published:** 2025-02-19

**Authors:** Conor C Carey, Eve M Creedon, Fionn Molloy, Morgan Lewis, Ben Leen Smith, Elaine K McCarthy

**Affiliations:** 1 School of Food and Nutritional Sciences, University College Cork, Cork, Ireland; 2 INFANT Research Centre, University College Cork, Cork, Ireland

**Keywords:** sport, exercise, food choice, athlete, sensory, food, food skills, physical activity

## Abstract

**Background:**

Food choice determinants are generally influenced by sociocultural, sensory, nutritional, and economic factors, among others. For athletes, these choices are further complicated by additional sporting and nutritional demands. Few large-scale studies have investigated the factors affecting the food choice of athletic populations, particularly across sporting categories and competition levels.

**Objectives:**

The objective of this study is to explore the determinants of food choice and the factors that influence it in a large cohort of athletes and active individuals.

**Methods:**

A self-administered online survey of athletes and active individuals (aged >18 y) was conducted using the previously validated Athlete Food Choice Questionnaire that comprised thirty-six statements across 9 food choice categories. Participants were eligible if taking part in competitive sport or structured physical activity sessions at least twice weekly.

**Results:**

In this cross-sectional sample (*n* = 1145), 62% (707) were female, the median (interquartile range) age was 26 y (21–40), with 7 h/wk (5–10) of exercise training/competition. “Sensory Appeal” was identified as the primary driver of food choice (mean rank [MR] = 7.46). This was followed by “Food and Health Awareness” (MR = 6.78) and “Performance” (MR = 6.65). Food values and beliefs were the least influential food choice category (MR = 2.06). Key predictors of food choice in this cohort included gender, competition level, sport type, and nutrition knowledge. For example, the “nutritional attributes of a food” were significantly less influential on team sport athletes’ food choice compared with endurance athletes [odds ratio (95% confidence interval): 0.552 (0.375, 0.813), *P* = 0.003].

**Conclusions:**

In one of the largest investigations to date, sensory appeal was the predominant influence on food choice across all sport types and competition levels. “Food and health awareness” and “performance” related factors also had considerable impact, although food values and beliefs were least influential.

## Introduction

The dynamics of human food consumption results in a complex behavioral system. An initiative to map this system has resulted in the determinants of nutrition and eating (DONE) framework [1], with 3 core outcomes identified: food choice, eating behavior, and dietary intake. Although considerable research to date has investigated the eating behavior and dietary intakes of athletes and active individuals, food choice has been comparatively under-researched despite its considerable impact on the diet and performance of this population group.

Food choice encompasses nutrition and eating-related outcomes that precede the actual consumption of a food. A conceptual model of food choice in the general population has highlighted that food choice is directly influenced by a matrix of food-internal factors (sensory and perceptual features), food-external factors (social and physical environment), personal-state factors (biological, physiological, habits, and psychological influences), cognitive factors (knowledge and skills, attitudes, and consequences), and sociocultural factors (cultural, economic and political variables) [2]. For athletes, these choices are further complicated by altered physiological demands as well as additional sporting and nutritional demands [3]. It has been shown that adults make >200 food-related choices per day, but given the greater nutritional demands placed on those undertaking exercise regimes, athletes are likely required to make even more food choices [4]. By understanding the many influences on this decision matrix, more tailored nutritional advice can be developed for differing athletic population groups, particularly those at risk of inadequate decision-making processes surrounding food.

A recent systematic scoping review investigating food choice in athletes identified a total of 15 research studies [5]. Seven studies used small-scale qualitative-based methods including focus group or interview-based methods, whereas the remaining 8 studies employed an observational, mostly survey-based design. This review concluded that food choice in athletes is clearly impacted by competition season, level of experience, sports culture, cultural background, nationality, sex of the athlete, and the food environment. Small-scale qualitative studies identified the importance of rules, routines [6], sporting characteristics [7], and body image [8] to the food choices of athletes.

Observational studies investigating food choices among mixed sporting groups often utilize small sample sizes (for example, *n* = 131 and *n* = 156), complicating analyses by sport type and competition level [9,10]. The only large-scale study (*n* = 769) to date, conducted in the “Athletes Village” dining hall during the Commonwealth Games, used an unvalidated tool and focused solely on elite, in-competition athletes [11]. This limited scope does not reflect broader food choice behaviors during training or among non-elite athletes.

The development and validation of the Athlete Food Choice Questionnaire (AFCQ) is a significant advancement, offering a reliable tool across various sports and competition phases [10]. However, its application has been limited, necessitating broader use to fully understand the determinants of food choice [9,10,12]. This study aims to be the largest investigation into the factors affecting athletes’ food choices, employing a validated research tool. It seeks to explore how various sporting and nonsporting factors influence these determinants, addressing the current gaps in research and providing comprehensive insights into athletes’ food choices.

## Methods

### Overarching study design

This cross-sectional study was conducted via a self-administered, online survey evaluating the factors that influence the food choice of athletes and active individuals in Ireland. This survey was delivered using the online survey platform Qualtrics (Qualtrics, Utah, USA).

The questionnaire included twenty-three questions relating to participant demographics, sporting and dietary practices, as well as the validated AFCQ and took ∼10 min to complete. The AFCQ comprises thirty-six statements divided into 9 categories where athletes rate the frequency that their food choice is affected on a 5-point Likert scale labeled from never (1) to always (5). This questionnaire has been validated for use in various population groups in an online setting [9,12]. The categories consist of the following: nutritional attributes of the food, emotional influences, food and health awareness, influence of others, usual eating practices, weight control, food values and beliefs, sensory appeal, and performance.

Ethical approval for this study was provided by the University College Cork Social Research Ethics Committee (SREC Log 2023-260) before the conduction of any study procedures.

### Recruitment

Participants in this study were required to be aged over 18 y, residing in Ireland, and engaged in competitive sports or structured physical activities at least twice weekly. Participants were recruited for this survey via various social media platforms, posters, word of mouth, and personal networks. Participants were provided with an information sheet at the beginning of the survey, outlining what the research involved and its purpose, and following this, they were asked to provide their informed consent before participation in the study. Participation was entirely voluntary and anonymous, with participants free to decline to answer specific questions or to withdraw from the study at any point. Participants who provided an incomplete response were given 7 d to complete their response before response closure. To be included in the analysis, participants were required to have completed a minimum of 75% of the survey questions. Data collection for this study took place between January and February 2024.

### Data analysis

Data were analyzed using IBM SPSS Statistics V28.0. Statistical significance was set at *P* < 0.05. Kolmogorov–Smirnov was used to assess data distribution, with data presented as median [interquartile range (IQR)] unless otherwise stated. Friedman One-Way Repeated Measure Analysis of Variance by Ranks was used to assess the importance of food choice determinants at total sample level. Ordinal logistic regression (OLR) was used to assess the impact of predictors including age, gender, country of origin, education level, living situation, nutrition knowledge, sport type, competition level, and training volume on each food choice category. A test of parallel lines was used to assess proportional odds. Results were presented as odds ratios (OR) with corresponding 95% confidence intervals (CI). Participants were only included in the OLR analysis if they had completed all questions related to that specific analysis, including questions related to both food choice category and predictors; missing data were excluded from the analysis. As such, a smaller sample size is present in each of these analyses.

The matrix of food choice categories and predictors as assessed by the Friedman test and OLR results is presented as one complete decision matrix via a Sankey diagram, a visualization tool used across multiple fields [13,14]. The initial node represents the overall food choice decision. The second level consists of 9 nodes, each representing a food choice category. The size of each node was proportional to the mean rank (MR) derived from the Friedman test. The third level includes nodes for significant predictors identified through OLRs. The size of these nodes was determined by the sum of the parameter estimates of significant interactions in OLR for that food choice category, adjusted proportionally to the importance of the corresponding category’s MR from the Friedman test.

## Results

### Participants

One thousand, one hundred and forty-five participants completed this online survey and were deemed eligible for inclusion (Supplemental Figure 1). Participant demographics and sporting characteristics are presented in Table 1. Thirty-eight percent of participants reported identifying as male, 62% identified as female, although <1% identified as other or preferred not to disclose their gender. Participants ranged in age from 18 to 72 y, with a median (IQR) age of 26 y (21–40).

**TABLE 1:** Participant demographic and sporting characteristics (*N* = 1145).

	Total 1145 (100)	Male, 430 (38)	Female, 707 (62)	Other/prefer not to say, 8 (1)
Country of origin				
Ireland	1001 (87)	373 (87)	619 (88)	6 (75)
United Kingdom	56 (5)	20 (5)	36 (5)	—
United States	21 (2)	4 (1)	16 (2)	1 (13)
Other European	36 (3)	16 (4)	19 (3)	1 (13)
Rest of the world	33 (3)	17 (4)	16 (2)	—
Sport category				
Endurance	468 (41)	182 (42)	280 (40)	4 (50)
Intermittent/team	347 (30)	157 (37)	186 (26)	3 (38)
Strength/power	214 (19)	53 (12)	160 (23)	1 (13)
Combat	24 (2)	15 (4)	12 (2)	—
Artistic	18 (2)	1 (<1)	17 (2)	—
Racquet	17 (2)	5 (1)	12 (2)	—
Other	54 (5)	17 (4)	37 (5)	—
Competition level				
Not competing	370 (32)	107 (25)	261 (37)	2 (25)
Local	398 (35)	161 (37)	234 (33)	1 (13)
Regional	108 (9)	34 (8)	74 (11)	—
University	81 (7)	40 (9)	37 (5)	3 (38)
National	127 (11)	64 (15)	61 (9)	2 (25)
International	57 (5)	23 (5)	34 (5)	—
Phase of competition				
Off season	89 (8)	28 (7)	60 (9)	1 (13)
Preseason	357 (31)	149 (35)	205 (29)	1 (13)
In-season	326 (28)	145 (34)	176 (25)	4 (50)
Training volume (h/wk)	7 (5–10)	8 (5–10)	6 (5–9)	8 (3–8)
Age (y)	26 (21–40)	25 (21–39)	28 (21–40)	20 (20–35)

Presented as *n* (%) or median (IQR) as appropriate.

Abbreviation: IQR, interquartile range

### Food choice determinants

**FIGURE 1:**
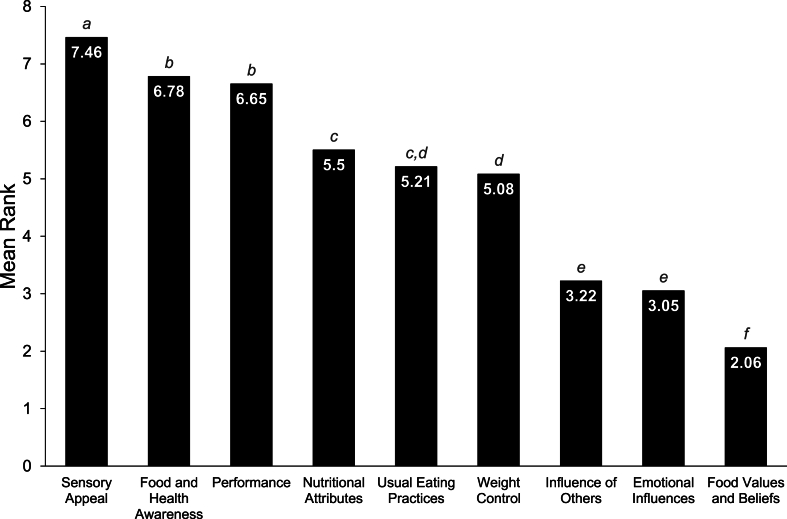
Mean ranks of food choice categories prioritized by athletes and active individuals (*n* = 956). Different superscript letters (a, b, c, etc.) denote significant differences (*P* < 0.05) as assessed by Friedman One-Way Repeated Measure Analysis of Variance by Ranks with a Bonferroni correction for multiple analyses (*n* = 946).

“Sensory appeal” was highlighted as the most influential factor in food choices, followed by “food and health awareness” and “performance.” Pairwise comparisons identified that sensory appeal was ranked significantly more important than all other food choice categories (MR = 7.46, all *P* < 0.01*).* There was no significant difference between “performance” (MR = 6.65) and “food and health awareness” (MR = 6.78, *P* > 0.05); however, both were ranked higher than all categories other than “sensory appeal” (all *P* < 0.01). Full rankings of food choice categories are presented in Figure 1.

**FIGURE 2:**
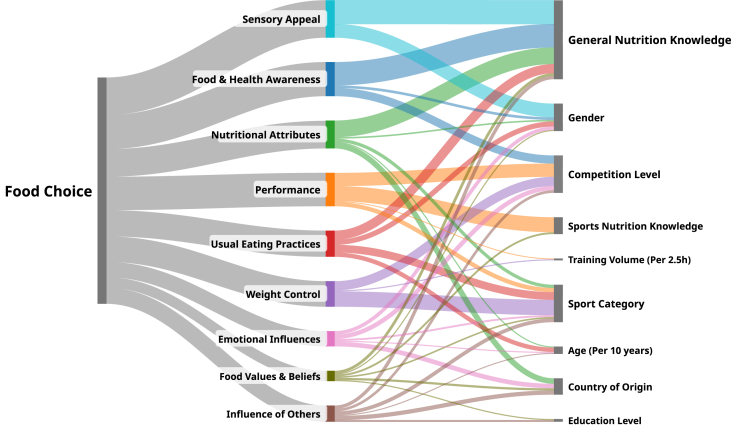
Sankey diagram of athlete food choices. The first node represents the overall food choice. The size of the second-tier nodes is proportional to mean rank scores of each food choice category from the Friedman test. The third-tier nodes indicate significant predictors identified by ordinal logistic regression.

Each food choice category was shown to be significantly influenced by various sporting and nonsporting predictors via OLR analysis. These interactions are presented via a Sankey diagram (Figure 2).

The individual statements that were ranked the most influential on food choice were “the taste of the food” (MR = 28.29) and “the flavor of the food” (MR = 27.06), followed by “the nutritional content of the food” (MR = 26.10) and “my ability to cook for myself” (MR = 25.18). The least influential statements were “my religious food beliefs” (MR = 3.77) and “how angry I feel” (MR = 8.33). Full evaluation of individual statements is shown in Table 2.

**TABLE 2:** Individual food choice statements in order of mean rank (*N* = 956).

Statement	Category	Mean rank
The taste of the food.	Sensory appeal	28.29^a^
The flavor of the food.	Sensory appeal	27.06^a,b^
The nutritional content of the food. (protein, fat, carbohydrate)	Nutritional attributes	26.10^b,c^
My ability to cook for myself.	Food and health awareness	25.18^b,c,d^
My need to feel energetic for training & competing.	Performance	24.71^c,d^
My awareness of the foods I have already consumed today.	Food and health awareness	24.39^c,d,e^
My need to fuel my body for competition.	Performance	23.58^d,e,f^
My need to fuel my body for recovery.	Performance	23.34^d,e,f^
My knowledge of nutritious foods.	Food and health awareness	22.64^e,f,g^
How familiar the food is to me.	Usual eating practices	22.15^f,g,h^
The sensory appeal of available foods.	Sensory appeal	21.94^f,g,h,i^
How convenient the food is to prepare/eat.	Food and health awareness	21.84^f,g,h,i^
My ability to plan my foods ahead.	Food and health awareness	21.70^f,g,h,I,j^
If the food might make my gut feel uncomfortable when training or competing (that is, stomach upset).	Performance	20.75^g,h,i,j,k^
The availability of food close to where I am.	Usual eating practices	20.44^h,I,j,k^
How much money I have to spend on food.	Usual eating practices	20.06^i,j,k^
The natural content of the food.	Nutritional attributes	20.05^i,j,k^
If the food is beneficial for my weight goal.	Weight control	19.89^j,k,l^
If I am trying to lose or gain weight.	Weight control	19.77^k,l,m^
How happy I am with my current weight/body image.	Weight control	19.23^k,l,m,n^
The foods that I’ve grown up eating.	Usual eating practices	18.08^l,m,n^
The presence of vitamins and minerals in the food.	Nutritional attributes	17.87^m,n^
Whether the food is a wholefood.	Nutritional attributes	17.6^n,o^
The health or nutrition claims about the food.	Nutritional attributes	17.37^n,o^
What my family is eating.	Influence of others	15.86^o,p^
Whether I am in the off season (no competitions or intense training for a period of time).	Weight control	14.94^p,q^
How stressed I feel.	Emotional influences	14.6^p,q,r^
How sad I feel.	Emotional influences	13.17^q,r,s^
My cultural style of eating (e.g., S. American, Indian, Western).	Usual eating practices	12.97^r,s^
If the food is sustainably produced.	Food values and beliefs	12.38^s^
What other athletes in my sport are eating.	Influence of others	12.29^s^
Eating to comfort my emotions.	Emotional influences	12.09^s^
If the food aligns with my values for animal welfare (i.e., no animal products/vegan, cruelty-free raised animals).	Food values and beliefs	11.47^s,t^
What my friends are eating.	Influence of others	10.12^t,u^
How angry I feel.	Emotional influences	8.33^u^
My religious food beliefs.	Food values and beliefs	3.77^v^

Different superscript letters (a, b, c, etc.) denote significant difference (*P* < 0.05) as assessed by Friedman One-Way Repeated Measure Analysis of Variance by Ranks with a Bonferroni correction for multiple analyses.

### Sensory appeal

**FIGURE 3:**
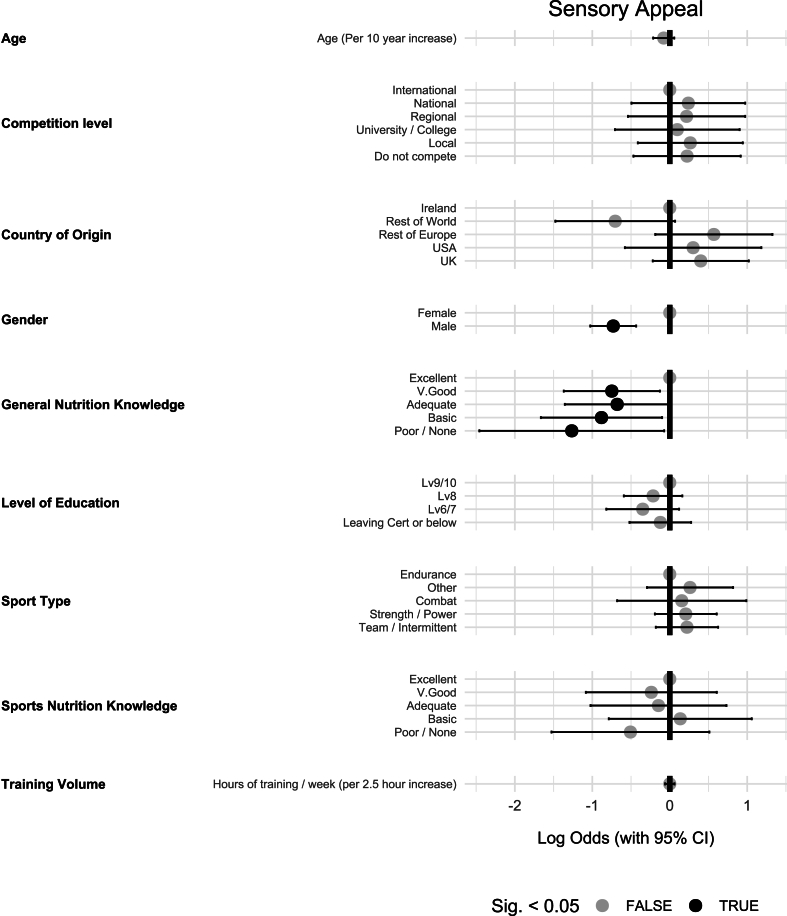
Ordinal logistic regression results investigating the potential influence of various predictors on the importance of sensory appeal in athlete food choice (*n* = 689).

OLR analysis investigating the impact of predictors on the importance of “sensory appeal” on food choice identified 2 significant predictors: gender and general nutrition knowledge (Figure 3). Athletes identifying as male were significantly less likely than female athletes to be influenced by the sensory appeal of food [OR (95% CI): 0.48 (0.36, 0.65), *P* < 0.01]. Athletes with excellent general nutrition knowledge were less influenced by sensory appeal compared with those with poor or no knowledge [OR (95% CI): 0.28 (0.09, 0.93), *P* = 0.04], basic knowledge [OR (95% CI): 0.41 (0.19, 0.91), *P* = 0.03], adequate knowledge [OR (95% CI): 0.51 (0.26, 0.99), *P* = 0.049], and very good knowledge [OR (95% CI): 0.47 (0.25, 0.88), *P* = 0.02].

### Food and health awareness

**FIGURE 4:**
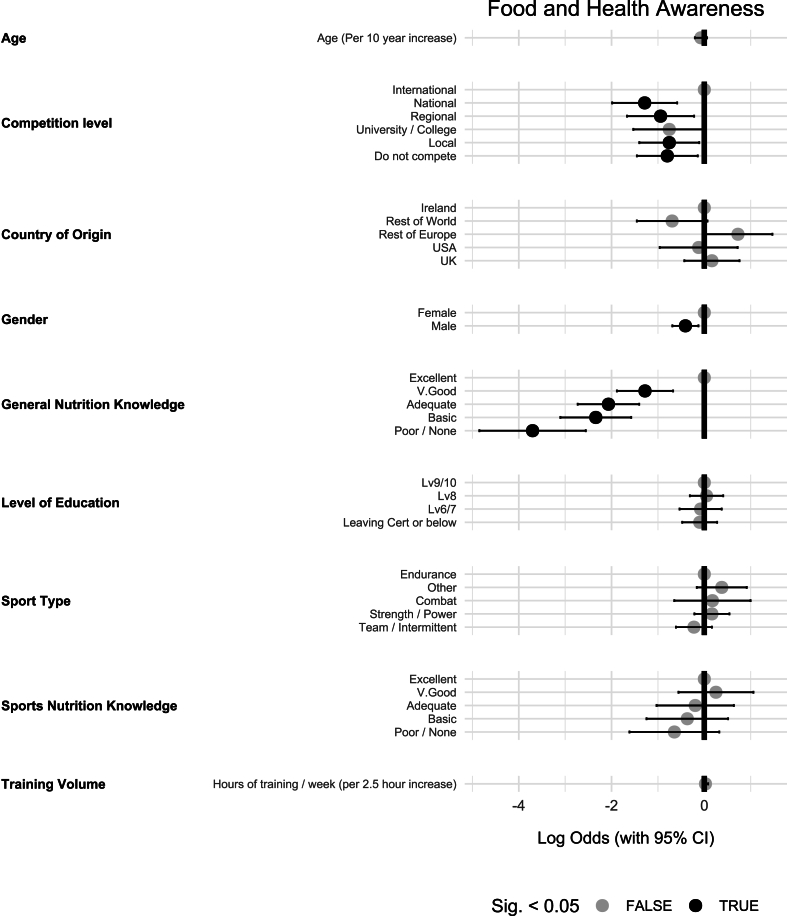
The influence of various predictors on the importance of Food and Health Awareness in athlete food choice (*n* = 734).

The predictors that influence the importance of “food and health awareness” as identified by OLR are presented in Figure 4. Male athletes’ food choices were significantly less likely than females to be influenced by factors of “food and health awareness” [OR (95% CI): 0.67 (0.50, 0.88), *P* < 0.01]. When compared with international competitors, all other levels of competition exhibited significantly lower odds of food and health awareness factors influencing their food choices. Specifically, athletes who do not currently compete [OR (95% CI): 0.45 (0.23, 0.87), *P* = 0.02], local competitors [OR (95% CI): 0.47 (0.25, 0.90), *P* = 0.02], regional competitors [OR (95% CI): 0.39 (0.19, 0.80), *P* = 0.01], and national competitors [OR (95% CI): 0.28 (0.14, 0.56), *P* < 0.01], all had significantly lower odds compared with international athletes. University-level athletes however did not significantly differ from international competitors in the influence of food and health awareness on their food choices.

Athletes with none/poor knowledge [OR (95% CI): 0.03 (0.01, 0.08), *P* < 0.01], basic knowledge [OR (95% CI): 0.10 (0.05, 0.21), *P* < 0.01], adequate knowledge [OR (95% CI): 0.13 (0.07, 0.25), *P* < 0.01], and very good knowledge [OR (95% CI): 0.28 (0.15, 0.51), *P* < 0.01], all exhibited lower odds of “food and health awareness” influencing their food choices compared with those with excellent knowledge.

### Performance

**FIGURE 5:**
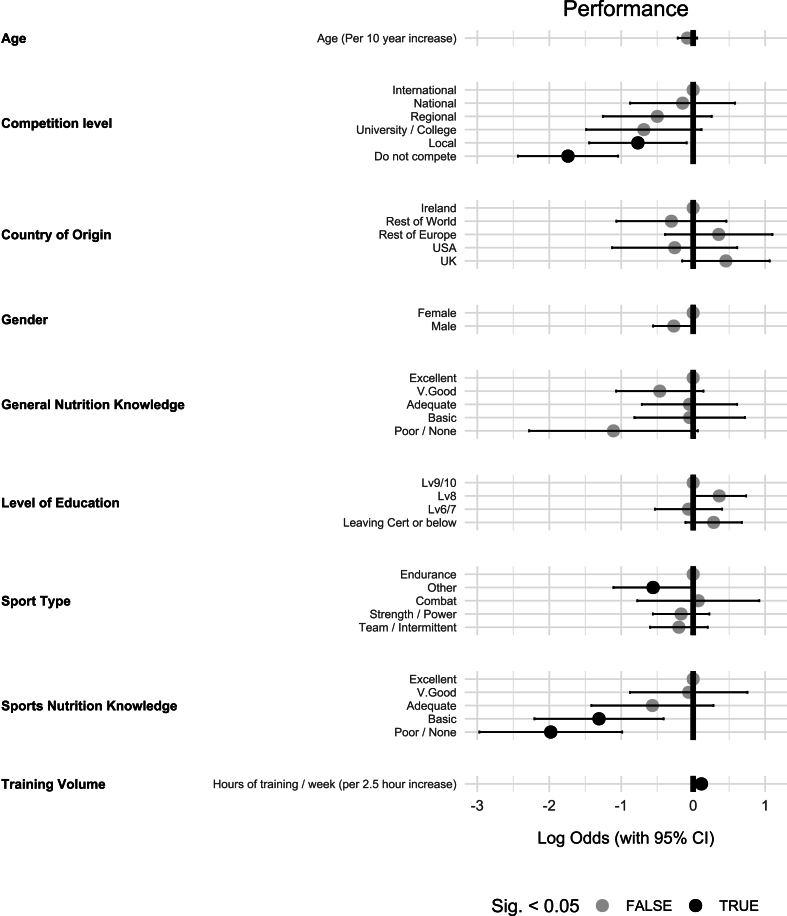
The influence of various predictors on the importance of the performance-related factors of food on athlete food choice (*n* = 687).

The impact of various predictors on the influence of “performance” related statements is presented in Figure 5. There was a significant positive relationship between the hours of training per week and the influence of “performance”-related statements. For each additional 2.5 h of training per week, the odds of “performance” influencing food choice increased significantly [OR (95% CI): 1.12 (1.06, 1.19), *P <* 0.01].

Athletes in endurance sports were more likely to have their food choice influenced by “performance”-related statements in comparison with athletes in the “other” sports category [OR (95% CI): 0.57 (0.33, 0.99), *P* = 0.048]. This category consisted of sports that did not fall into any of endurance, team sport, strength and power sports and combat sports categories. Both athletes who do not currently compete [OR (95% CI): 0.18 (0.09, 0.35), *P* < 0.01], and athletes competing at the local level [OR (95% CI): 0.46 (0.24, 0.91), *P* = 0.03] exhibited significantly lower odds of being influenced by “performance”-related statements compared with international competitors.

Athletes who reported their sports nutrition knowledge as none/poor [OR (95% CI): 0.14 (0.05, 0.37), *P* < 0.01] or basic [OR (95% CI): 0.27 (0.11, 0.66), *P<* 0.01] were significantly less likely to be influenced by “performance”-related statements compared with those with excellent knowledge.

### Nutritional attributes

**FIGURE 6:**
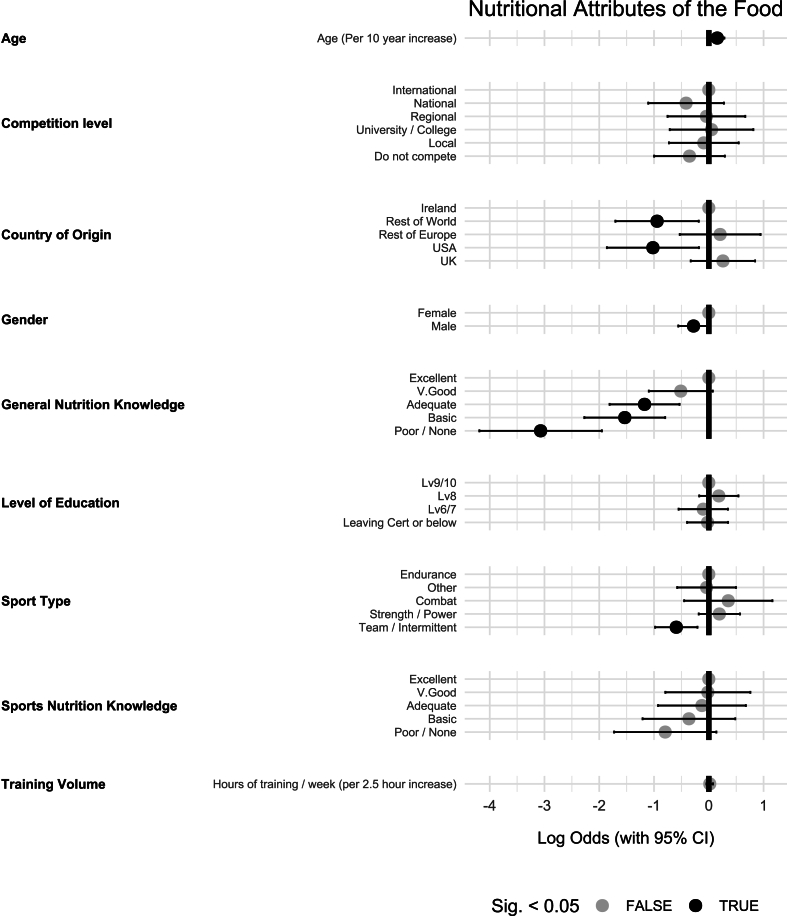
The influence of various predictors on the importance of the nutritional attributes of food on athlete food choice (*n* = 747).

The influence of various predictors on the importance of the “nutritional attributes of a food” on athletes’ food choices is presented in Figure 6. For every 10-y age increase, the “nutritional attributes” were more likely to impact the food choice of the athlete [OR (95% CI): 1.17 (1.02, 1.33), *P* = 0.02]. Males were significantly less likely to be influenced by the “nutritional attributes of a food” than females [OR (95% CI): 0.76 (0.57, 0.99), *P* = 0.048]. The “nutritional attributes of a food” also had less influence on the food choice of athletes who were born in the USA [OR (95% CI): 0.36 (0.16, 0.84), *P* = 0.02] or rest of the world (excluding Ireland, UK, USA, and rest of Europe) [OR (95% CI): 0.39 (0.18, 0.83), *P* = 0.02] when compared with athletes born in Ireland.

It was also found that “nutritional attributes” influenced food choice significantly more when the athlete had greater general nutrition knowledge. Those with poor/no knowledge [OR (95% CI): 0.05 (0.02, 0.14), *P >* 0.01], basic knowledge [OR (95% CI): 0.22 (0.10, 0.45), *P <* 0.01], and adequate knowledge [OR (95% CI): 0.30 (0.16, 0.59), *P <* 0.01] were all significantly less likely to be influenced compared with those with excellent knowledge. Compared with endurance athletes, team sport athletes were significantly less likely to be influenced by statements in the “nutritional attributes of a food” category [OR (95% CI): 0.55 (0.38, 0.81), *P* < 0.01].

### Usual eating practices

For every 10-y age increase, an athlete’s “usual eating practices” had less of an impact on their food choice [OR (95% CI): 0.69 (0.60, 0.79), *P* < 0.01] (Supplemental Figure 2). Males were also less influenced by statements related to “usual eating practices” than females [OR (95% CI): 0.64 (0.48, 0.86), *P <* 0.01]. Those with basic general nutrition knowledge were significantly more likely to be influenced by “usual eating practices” than those with excellent general nutrition knowledge [OR (95% CI): 2.21 (1.03, 4.71), *P* = 0.04].

### Emotional influences

As an athlete gets older (for every 10 y), their food choice is significantly less likely to be influenced by statements in the “emotional influences” category [OR (95% CI): 0.76 (0.66, 0.86), *P <* 0.01] (Supplemental Figure 3). Males are also significantly less likely to be affected by “emotional influences” than females [OR (95% CI): 0.39 (0.29, 0.52), *P* < 0.01]. Team sport athletes are less likely to be influenced by emotional eating when compared with endurance athletes [OR (95% CI): 0.61 (0.41, 0.90), *P* = 0.01]. Local [OR (95% CI): 2.76 (1.45, 5.25), *P <* 0.01], regional [OR (95% CI): 2.49 (1.22, 5.08), *P* = 0.01], and university-level athletes [OR (95% CI): 3.21 (1.48, 6.94), *P* < 0.01] were significantly more impacted by their emotions when choosing the foods they eat when compared with international level athletes.

### Weight control

As training volume increases, “weight control” is significantly more likely to influence an athlete’s food choice [OR (95% CI): 1.09 (1.03, 1.16), *P* = 0.01] per 2.5 h/wk increase (Supplemental Figure 4). Those competing in combat sports were also significantly more likely to be influenced by statements related to “weight control” compared with endurance athletes [OR (95% CI): 3.47 (1.52, 7.93), *P <* 0.01].

### Influence of others

As athletes age, they are significantly less likely to have their food choices influenced by others [OR (95% CI): 0.73 (0.63, 0.83), *P* < 0.01] for every 10-y older (Supplemental Figure 5). Country of origin was also shown to significantly influence the chance of food choice being influenced by others, with those born in the rest of the world [OR (95% CI): 0.25 (0.12, 0.52), *P <* 0.01], and those born in the rest of Europe [OR (95% CI): 0.41 (0.19, 0.89), *P* = 0.02], significantly less likely to be influenced by others than those born in Ireland. Strength and power athletes [OR (95% CI): 0.63 (0.43, 0.92), *P* = 0.02] and combat athletes [OR (95% CI): 0.30 (0.13, 0.68), *P <* 0.01] were also less likely to be influenced by others in their food choice compare with endurance athletes.

### Food values and beliefs

Food values and beliefs were on average the least influential food choice category in the decision-making of athletes (MR = 2.06). Individual statements in this category were among the lowest-ranked statements including those related to religious beliefs (MR = 3.77), animal welfare (MR = 11.47), and sustainability (MR = 12.38). The food choices of male athletes were significantly less influenced by statements in the “food values and beliefs” category compared with female athletes [OR (95% CI): 0.58 (0.44, 0.77), *P <* 0.01] (Supplemental Figure 6). Those with secondary education or lower were significantly less impacted by “food values and beliefs” in their food choice [OR (95% CI): 0.57 (0.38, 0.84), *P* < 0.01], when compared with athletes with master’s or higher level of education. Team sports athletes were also less influenced by “food values and beliefs” than their endurance counterparts [OR (95% CI): 0.46 (0.31, 0.69), *P* < 0.01].

## Discussion

This study is the most comprehensive investigation into the factors influencing athletes’ food choices to date. It uniquely explores and ranks the determinants of food choice, assessing how these factors interact with athletes’ sporting, sociocultural and demographic backgrounds. These findings highlight sensory appeals, such as taste and flavor, as the primary driver of an athletes’ food choices across all population groups, consistent with earlier studies emphasizing its importance in eating behavior [3]. Furthermore, performance-related factors (for example, fuel for training and recovery) and food and health awareness (for example, awareness of previously consumed food and cooking ability) also significantly impact food choices, particularly in those competing at higher levels, reinforcing the importance of these factors in dietary decision-making processes among athletes. The study also highlighted significant demographic influences, with gender and nutrition knowledge being crucial predictors for several important food choice categories.

Sensory preference is central to dietary patterns in the general population [15], and this study confirms its undiminished importance across all sports and competition levels. As such, nutritional advice given to athletes of all levels should be cognizant of the dietary preferences of athletes as adherence to advice is likely governed by the sensory appeal of the food, even if the athlete competes at an elite level. In contrast, a previous analysis of food choice determinants in the athletes’ village during 2 Commonwealth Games identified sensory factors to be ranked less influential than performance and health-related factors such as nutritional composition, stage of competition, time of day, and food familiarity [11]. It is possible that the importance of the sensory appeal of food may only be diminished in high-performance athletes during specific acute competition phases when dietary practice has a direct impact on competition performance. However, a limitation of the AFCQ tool is its omission of certain sensory aspects like aroma and texture, suggesting the need for a more comprehensive analysis to fully understand sensory appeal’s role. Additionally, data are needed to identify the common food preferences of athletes. Validated food preference assessment methods should be considered in athletic populations such as the Leeds food preference questionnaire [16,17].

Food and health awareness emerged as a top influencer of food choice, particularly the ability to cook, which may limit the consumption of certain food groups. A previous analysis of the cooking and food skills of Irish team sport athletes has shown that this population group has lower skill levels than other population groups including young Australian adults [18]. Knowledge of nutritious foods was also ranked as a highly influential determinant of food choice in athletes. Nutrition knowledge has been extensively researched [19]; multiple studies have shown that nutrition knowledge is poor among Irish athletes, although this has mainly focused on team sport athletes to date [[20], [21], [22]]. Previous research has shown in athletes with relative energy deficiency in sport (RED-S), nutrition knowledge interventions can have a positive impact on knowledge which translates to a modest impact on behavior over a 16-wk period [23].

Factors related to nutrition planning and the influence of previously consumed food were also ranked as important influencers on food choice in the food and health awareness category. In the cooking and food skills questionnaire administered previously to Irish team sport athletes, the skill of “planning meals ahead” was ranked as the third lowest rated skill of a list of 19 food skills [18]. A promising area for future research may be interventions involving planning techniques to enhance athletes’ dietary behavior. Although this has not been investigated in athletes, a previous planning intervention in nurses and midwives has shown positive impacts on dietary behavior [24]. There is considerable scope for interventions in cooking, food skills, nutrition knowledge, and food planning to improve eating behavior and dietary intake of athletes.

Feeling energetic and fueling for both competition and recovery were the most influential performance-related statements on food choice. This is in line with much of the research to date, including a recent scoping review highlighting that most of the 15 included studies reported the impact of performance-related factors on food choice [5]. A previous cross-sectional investigation of mixed athletic groups has shown that athletes’ most sought-after product centered around muscle recovery, followed closely by enhanced endurance performance [25].

“The nutritional content of the food (protein, fat, carbohydrate)” was ranked as the joint second most influential individual statement. This highlights the considerable importance placed on the macronutrient content of food by athletes, although the micronutrient content appeared to be significantly less influential in this cohort. This is worrying, as micronutrient deficiencies are linked with considerable detriments in performance, injury, and health [26]. Interestingly, non-European athletes were less influenced by the “nutritional attributes of a food” category compared with Irish-born athletes, despite all residing in Ireland, suggesting further research is needed on cultural influences. Sporting differences were also observed, with team sport athletes less influenced by the nutritional attributes of food than endurance athletes. This may be a result of the increased technical and tactical demands of team sports potentially reducing the perceived performance benefit of dietary behavior changes. Amateur team sports competitions in Ireland are particularly popular [27]; it is possible that nutrition may be less important compared with other cultures where professionalism is more prevalent. However, given the growth of research highlighting the impact of nutritional interventions on both cognitive and intermittent exercise performance, this may prove to be a considerable differentiating factor for those team sport athletes going forward [28,29].

Males were less impacted by usual eating practices than female athletes and aging also impacted the importance of usual eating practices, indicating that older athletes were more willing to branch out from historical norms. This indicates that when dealing with younger female athletes in particular, dietary advice should be particularly mindful of the athlete’s familiarity with foods and practices. Similar results were seen for emotional influences on food choice, with younger and female athletes particularly at-risk. This is concerning, given the high risk of disordered eating among this cohort [30]. Combat sport athletes were shown to be significantly more impacted by weight control factors than any other sport type.

Food values and beliefs were ranked as the lowest influence on an athlete’s food choice. This category relates to the impact of sustainability, animal welfare, and religious beliefs in particular. Despite current global trends toward more sustainable food systems [31], sustainability seems to minimally influence athletes’ food choices. This echoes the findings of another recent study that showed in the context of nutrition claims on sports nutrition products; sustainability was the least prioritized [25]. Athletes appear to be unlikely to be willing to compromise on the sensory and nutritional characteristics of food in favor of one that is more sustainably produced. Future consideration should focus on integrating sports nutrition within environmentally sustainable food systems, emphasizing the development of practices that align with the primary drivers of athletes’ food choices, such as sensory appeal and performance benefits, although incorporating new research findings that highlight the current minimal impact of environmental sustainability on athletes’ dietary decisions.

To the best of the authors’ knowledge, this is the largest investigation of food choice in the athletic population, with considerable representation of athletes across 32 different sporting disciplines, from all competition levels including elite international level as well as those undergoing noncompetitive, structured physical activity training exclusively. However, it must be cautioned that all data are self-reported via a previously validated questionnaire. Further research is required to assess whether self-reported food choice data are a true indicator of eating behavior and dietary intakes, the subsequent 2 outcomes of the DONE framework [1].

This study did not monitor or control for the current hunger status or time of day in which the questionnaire was completed. Importance of certain food choice statements may have been differently prioritized as a result of a subconscious effect hunger state at the time of completion. As sensory appeal was ranked as the most influential factor in an athlete’s food choice, further research is required in this space. Development of novel nutritional interventions should ensure that sensory analysis is carried out to assess its potential to be adopted in practice.

In conclusion, this study represents the largest investigation to date of food choice and its determinants in athletes and active individuals. Results highlight that the sensory appeal of food is the primary driver of food choice, followed by performance and food and health awareness factors. This study further highlights the complexity of food choice in athletic populations compared with the general public, because of the impact of sporting demands and sporting culture. In particular, performance considerations were rated as one of the most important determinants of food choice, and other determinants were shown to be significantly influenced by the sporting category, competition level and training volume of the athletes. Food and health awareness factors including cooking ability, nutrition knowledge, and food skills such as food planning may provide promising avenues where interventions may have considerable positive impacts on the dietary practice of athletes. The information presented in this study can be used to identify athlete groups at particular risk of poor dietary practices and that may benefit from targeted dietary interventions. One potentially at-risk group is young female athletes because of the increased influence of age and emotions on food choice compared with male athletes. Further research studies conducting retrospective food choice analysis related to specific eating events could provide valuable insights into how food choice interacts with dietary intake.

## Author contributions

The authors’ responsibilities were as follows – CCC, EKM: designed research (project conception, development of overall research plan, and study oversight), wrote paper (only authors who made a major contribution), and had primary responsibility for final content; CCC, EMC, FM, ML, BLS: conducted research (hands-on conduct of the experiments and data collection); CCC, ML: analyzed data or performed statistical analysis; and all authors: read and approved the final manuscript.

## Data availability

Data described in the manuscript, code book, and analytic code will be made available upon request.

## Funding

The authors reported no funding received for this study.

## Conflict of interest

The authors report no conflicts of interest.
