# Supplementary material for: Exploring Food Choice Influences in Athletes and Active Populations in Ireland: A Cross-Sectional Study
Source: Curr Dev Nutr. 2025 Feb 19;9(3):104568. doi: 10.1016/j.cdnut.2025.104568 (PMC11938079; doi:10.1016/j.cdnut.2025.104568)
Supplement: Multimedia component 1 [file mmc1.pdf]

Exploring food choice influences in athletes and active populations in Ireland: a cross-sectional study

Supplementary Materials

Conor C. Carey[1], Eve M. Creedon[1], Fionn Molloy[1], Morgan Lewis[1], Ben Leen Smith[1], Elaine K. McCarthy[1,2]*

[1] School of Food and Nutritional Sciences, University College Cork, Ireland

[2] INFANT Research Centre, University College Cork, Ireland.

Supplementary File 1

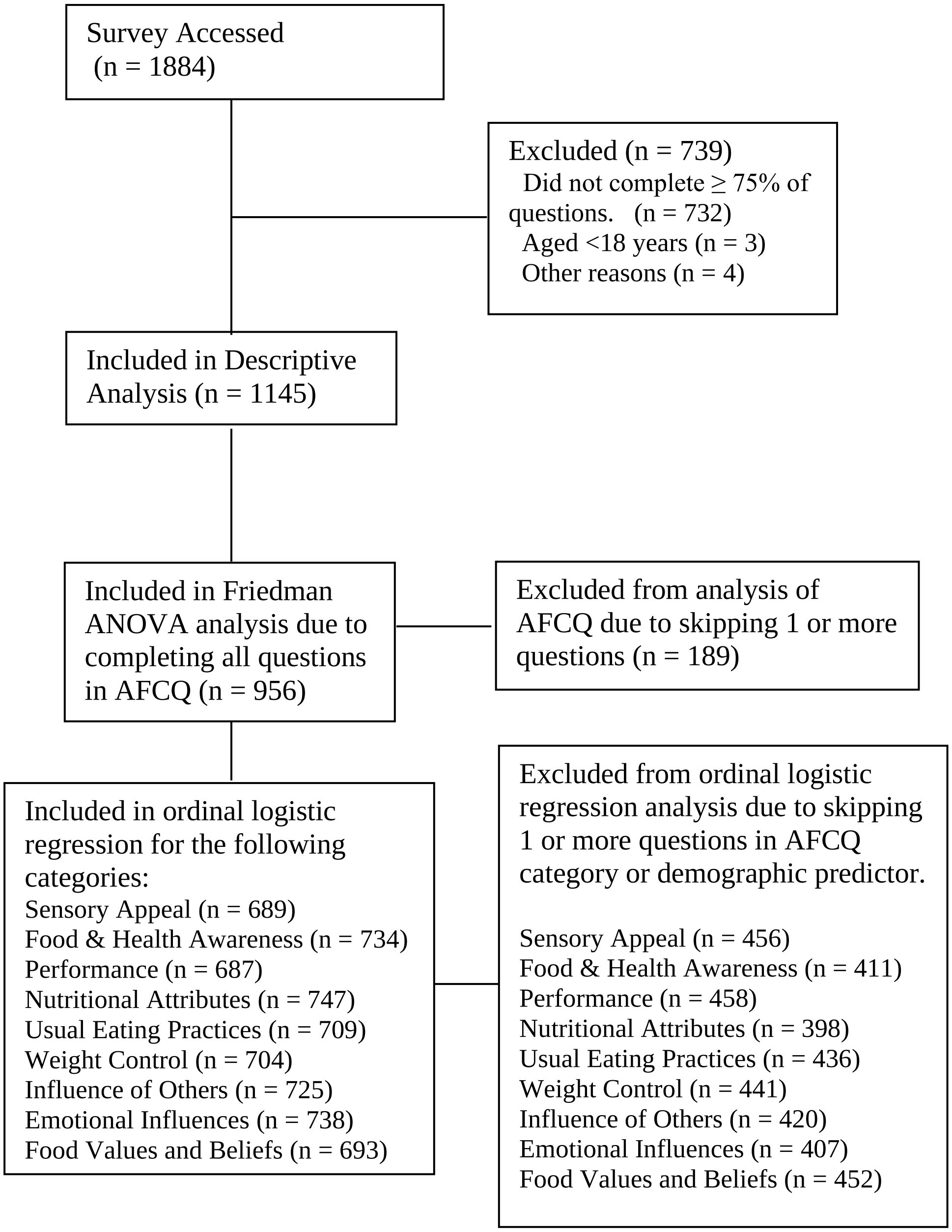

Supplementary File 2

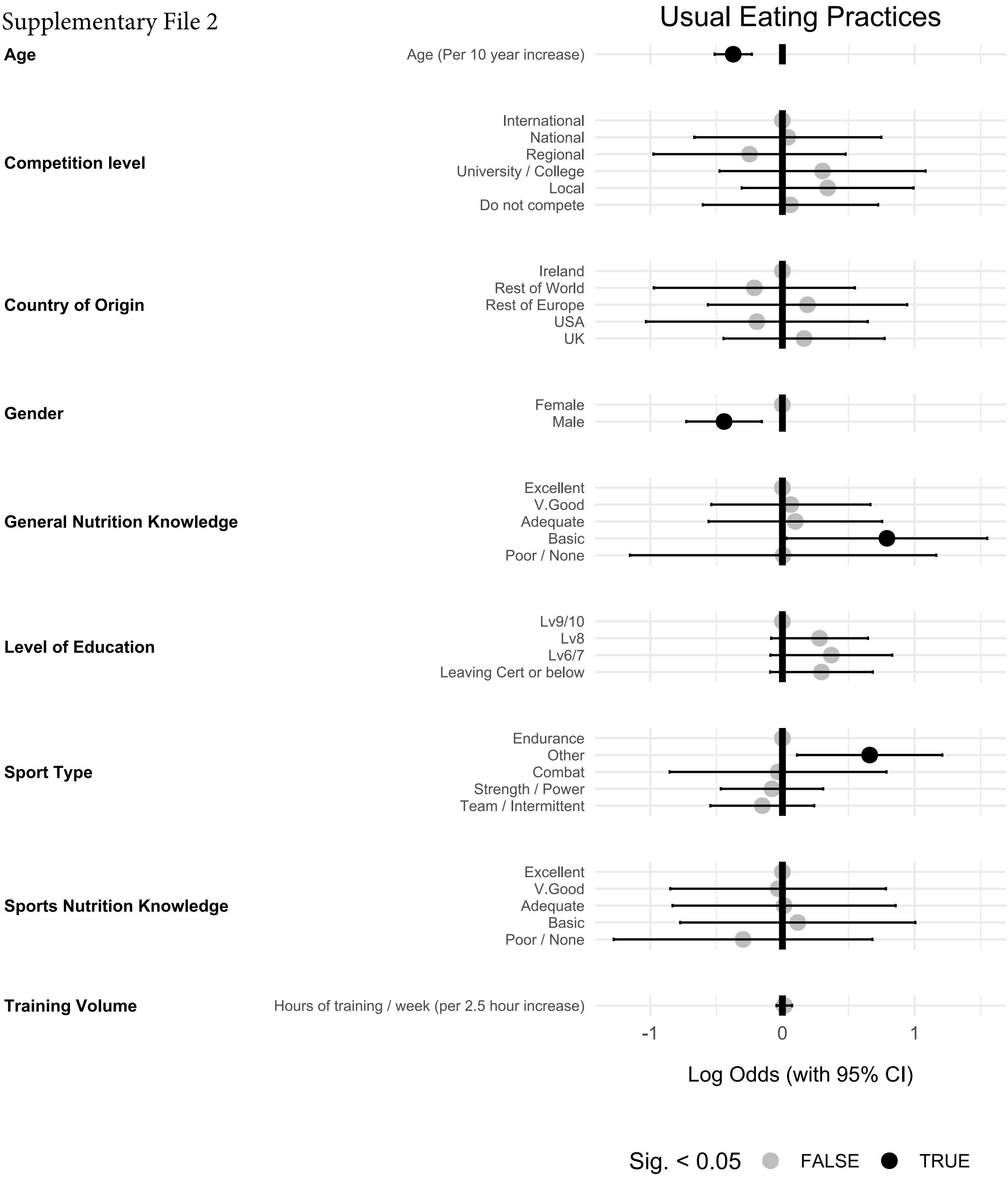

Supplementary File 3

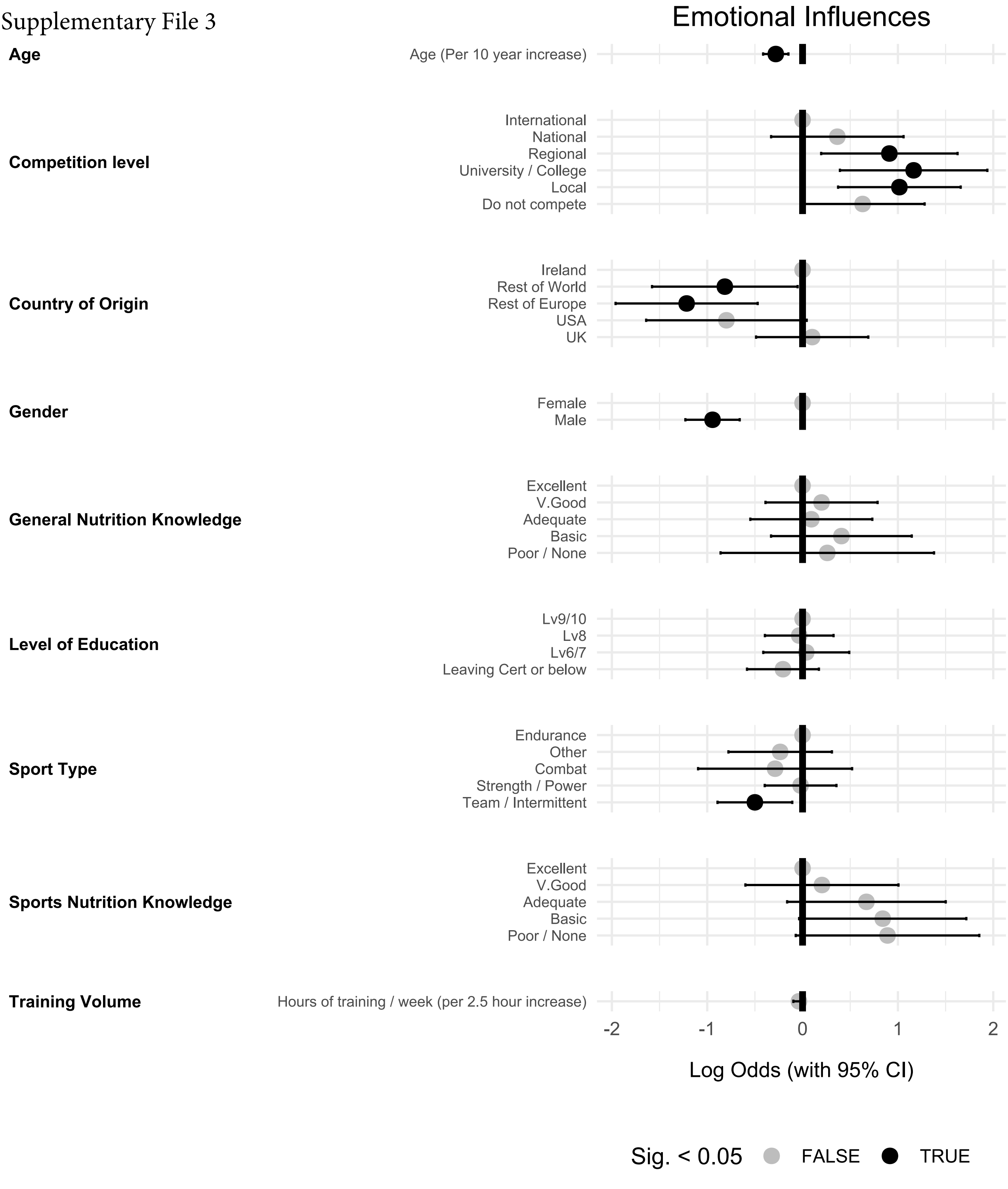

Supplementary File 4

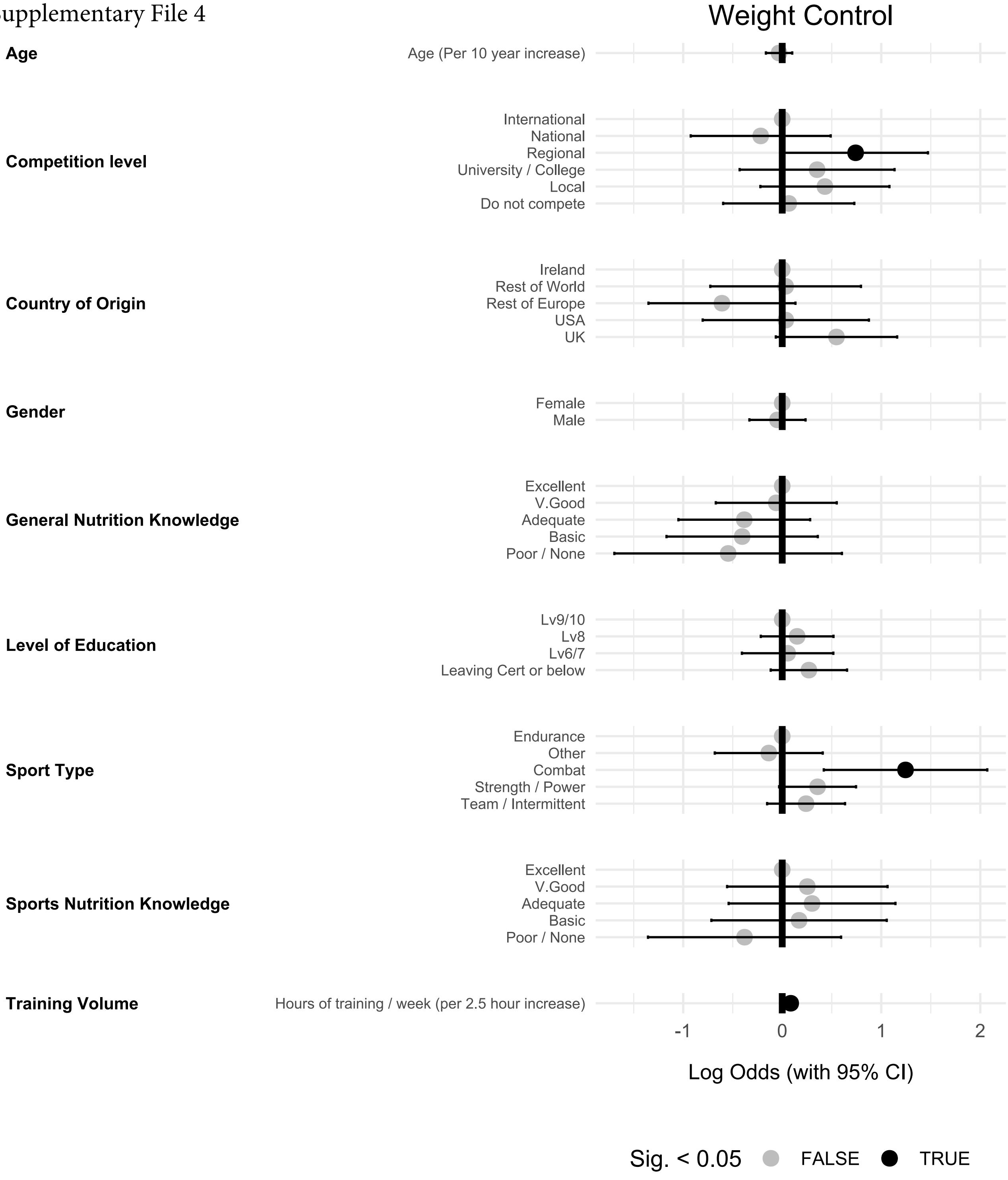

Supplementary File 5

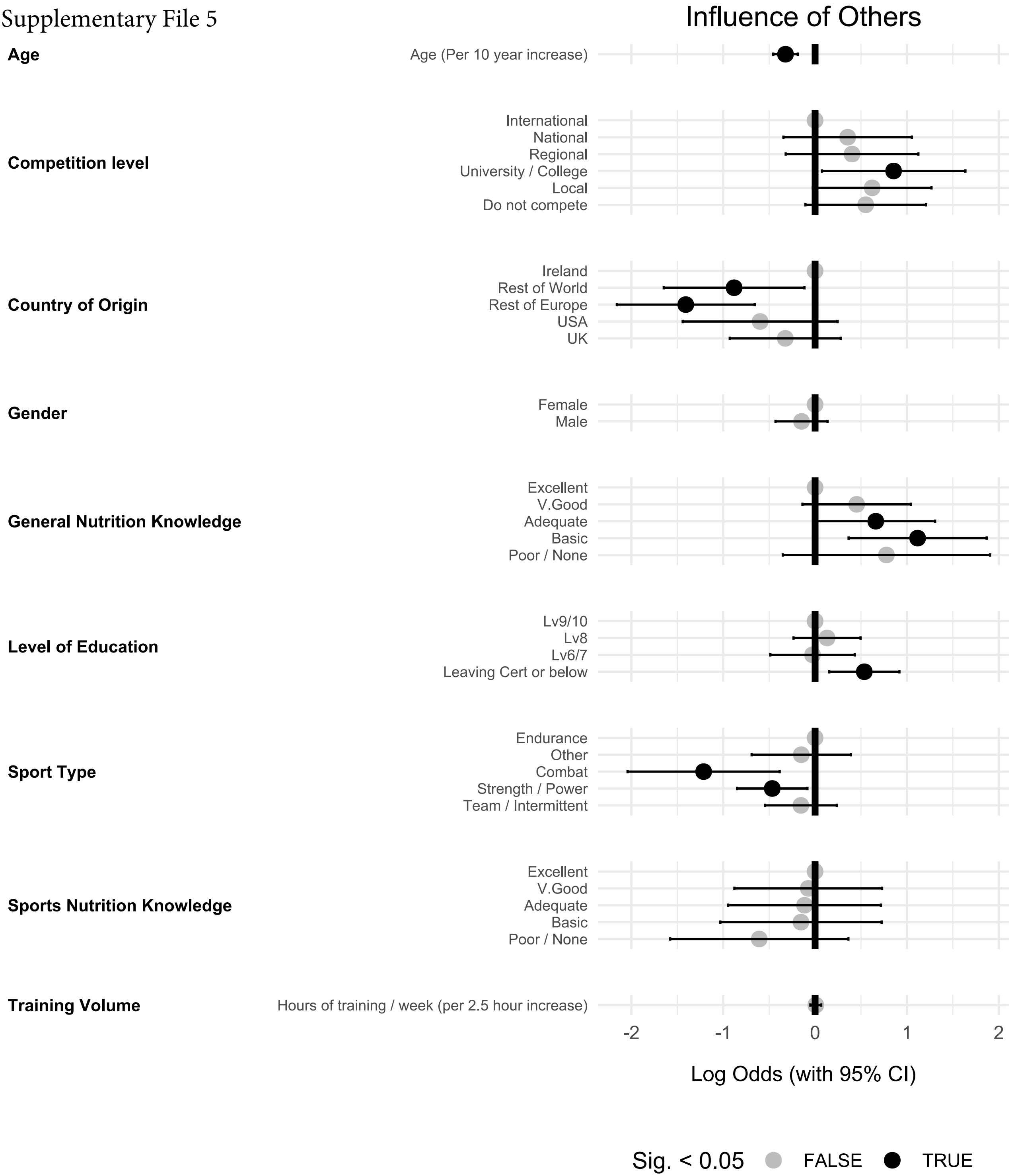

Supplementary File 6

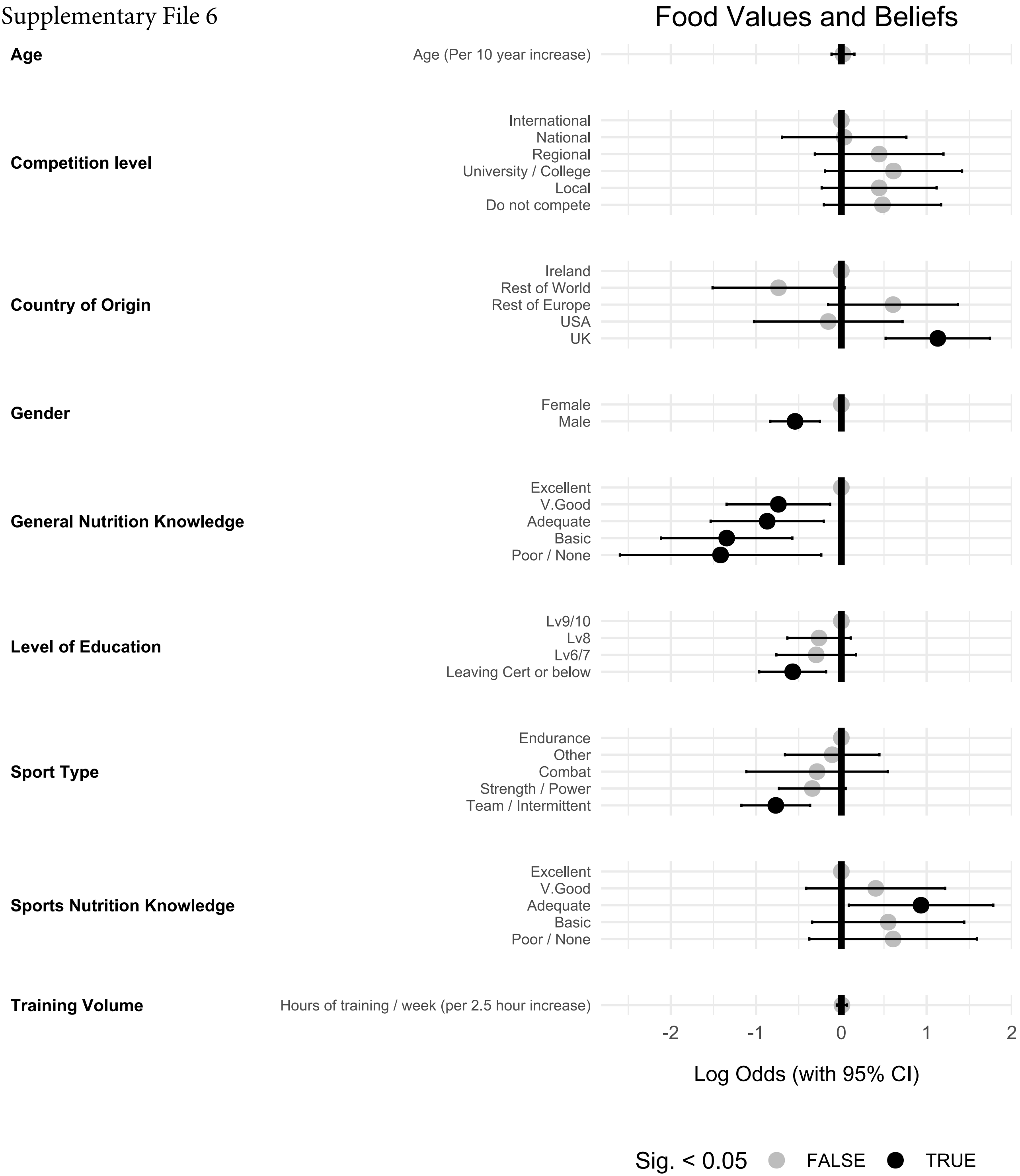

Supplementary File 7

Reporting checklist for observational studies in nutritional epidemiology.

Based on the STROBE-nut guidelines.

Instructions to authors

Complete this checklist by entering the page numbers from your manuscript where readers will find each of the items listed below.

Your article may not currently address all the items on the checklist. Please modify your text to include the missing information. If you are certain that an item does not apply, please write "n/a" and provide a short explanation.

Upload your completed checklist as an extra file when you submit to a journal.

In your methods section, say that you used the STROBE-nutreporting guidelines, and cite them as:

Lachat C, Hawwash D, Ocké MC, Berg C, Forsum E, Hörnell A, Larsson C, Sonestedt E, Wirfält E, Åkesson A, Kolsteren P, Byrnes G, De Keyzer W, Van Camp J, Cade JE, Slimani N, Cevallos M, Egger M, Huybrechts I. Strengthening the Reporting of Observational Studies in Epidemiology-Nutritional Epidemiology (STROBE-nut): An Extension of the STROBE Statement.

		Reporting Item	Page Number
**Title and abstract**			
Title	#1a	Indicate the study’s design with a commonly used term in the title or the abstract	1
None	#nut-1	State the dietary/nutritional assessment method(s) used in the title or in the abstract.	1
Abstract	#1b	Provide in the abstract an informative and balanced summary of what was done and what was found	1-2
**Introduction**			
Background / rationale	#2	Explain the scientific background and rationale for the investigation being reported	4

Objectives	#3	State specific objectives, including any prespecified hypotheses	4
**Methods**			
Study design	#4	Present key elements of study design early in the paper	5
Setting	#5	Describe the setting, locations, and relevant dates, including periods of recruitment, exposure, follow-up, and data collection	5&6
None	#nut-5	Describe any characteristics of the study settings that might affect the dietary intake or nutritional status of the participants, if applicable.	5&6
Eligibility	#6a	Cohort study: Give the eligibility criteria and the sources and methods of selection of participants. Describe methods of follow-up. Case-control study: Give the eligibility criteria and the sources and methods of case ascertainment and control selection. Give the rationale for the choice of cases and controls. Cross-sectional study: Give the eligibility criteria, and the sources and methods of selection of participants.	N/A
None	#nut-6	Report any particular dietary, physiologic, or nutritional characteristics that were considered when selecting the target population.	5
None	#6b	Cohort study: For matched studies, give matching criteria and number of exposed and unexposed. Case-control study: For matched studies, give matching criteria and the number of controls per case.	N/A
Variables	#7	Clearly define all outcomes, exposures, predictors, potential confounders, and effect modifiers. Give diagnostic criteria, if applicable	6
None	#nut-7.1	Clearly define foods, food groups, nutrients, or other food components (e.g., preparation method, taxonomical descriptors, classification, chemical form).	N/A
None	#nut-7.2	When calculating dietary patterns, describe the methods to obtain them and their nutritional properties.	N/A

Data sources and measurement	#8	For each variable of interest give sources of data and details of methods of assessment (measurement). Describe comparability of assessment methods if there is more than one group. Give information separately for for exposed and unexposed groups if applicable.	6
None	#nut-8.1	Describe the dietary assessment method(s) (e.g., portion size estimation, number of days and items recorded, how it was developed and administered, and how quality was ensured); report if and how supplement intake was assessed.	
None	#nut-8.2	Describe and justify food-composition data used; explain the procedure to match food composition with consumption data; describe the use of conversion factors, if applicable	N/A
None	#nut-8.3	Describe the nutrient requirements, recommendations, or dietary guidelines and the evaluation approach used to compare intake with the dietary reference values, if applicable	N/A
None	#nut-8.4	When using nutritional biomarkers, additionally use the STROBE-ME; report the type of biomarkers used and usefulness as dietary exposure markers	N/A
None	#nut-8.5	Describe the assessment of nondietary data (e.g., nutritional status and influencing factors) and timing of the assessment of these variables in relation to dietary assessment	5&6
None	#nut-8.6	Report on the validity of the dietary or nutritional assessment methods and any internal or external validation used in the study, if applicable	5
Bias	#9	Describe any efforts to address potential sources of bias	5&6
None	#nut-9	Report how bias in dietary or nutritional assessment was addressed (e.g., misreporting, changes in habits as a result of being measured, data imputation from other sources).	5&6
Study size	#10	Explain how the study size was arrived at	5

Quantitative variables	#11	Explain how quantitative variables were handled in the analyses. If applicable, describe which groupings were chosen, and why	6
None	#nut-11	Explain categorization of dietary/nutritional data (e.g., use of N-tiles and handling of nonconsumers) and the choice of reference category, if applicable.	N/A
Statistical methods	#12a	Describe all statistical methods, including those used to control for confounding	6
Subgroups and interactions	#12b	Describe any methods used to examine subgroups and interactions	6
Missing data	#12c	Explain how missing data were addressed	6
Loss to follow up	#12d	Cohort study: if applicable, explain how loss to follow-up was addressed. Case-control study: if applicable, explain how matching of cases and controls was addressed. Cross-sectional study: if applicable, describe analytical methods taking account of sampling strategy.	N/A
Sensitivity analysis	#12e	Describe any sensitivity analyses	N/A
None	#nut-12.1	Describe any statistical method used to combine dietary or nutritional data, if applicable.	N/A
None	#nut-12.2	Describe and justify the method for energy adjustments, intake modeling, and use of weighting factors, if applicable	N/A
None	#nut-12.3	Report any adjustments for measurement error (i.e., from a validity or calibration study).	N/A
**Results**			
Participants	#13a	Report numbers of individuals at each stage of study—eg numbers potentially eligible, examined for eligibility, confirmed eligible, included in the study, completing follow-up, and analysed. Give information separately for for exposed and unexposed groups if applicable.	7 & Additional File 1
Non-participation	#13b	Give reasons for non-participation at each stage	Additional File 1

Participant journey	#13c	Consider the use of a flow diagram	Additional File 1
None	#nut-13	Report the number of individuals excluded on the basis of missing, incomplete, or implausible dietary and nutritional data.	Additional File 1
Descriptive data	#14a	Give characteristics of study participants (eg demographic, clinical, social) and information on exposures and potential confounders. Give information separately for exposed and unexposed groups if applicable.	7
Missing data	#14b	Indicate number of participants with missing data for each variable of interest	Table 2, Figures 1, 3-6
Follow-up time	#14c	Cohort study: Summarise follow-up time (eg, average and total amount)	N/A
None	#nut-14	Give the distribution of participant characteristics across the exposure variables, if applicable; specify if food consumption for the total population or consumers only was used to obtain results	N/A
Outcome data	#15	Cohort study: report numbers of outcome events or summary measures over time. Case-control study: report numbers in each exposure category, or summary measures of exposure. Cross-sectional study: report numbers of outcome events or summary measures.	N/A
Main results	#16a	Give unadjusted estimates and, if applicable, confounder-adjusted estimates and their precision (eg, 95% confidence interval). Make clear which confounders were adjusted for and why they were included	N/A
Category boundaries	#16b	Report category boundaries when continuous variables were categorized	N/A
Relative and absolute risks	#16c	If relevant, consider translating estimates of relative risk into absolute risk for a meaningful time period	N/A
None	#nut-16	Specify if nutrient intakes are reported with or without the inclusion of dietary supplement intake, if applicable.	N/A

Other analyses	#17	Report other analyses done—eg analyses of subgroups and interactions, and sensitivity analyses	6
None	#nut-17	Report any sensitivity analysis (e.g., exclusion of misreporters or outliers) and data imputation, if applicable	N/A
**Discussion**			
Key results	#18	Summarise key results with reference to study objectives	14
Limitations	#19	Discuss limitations of the study, taking into account sources of potential bias or imprecision. Discuss both direction and magnitude of any potential bias.	18
None	#nut-19	Describe the main limitations of the data sources and assessment methods used and implications for the interpretation of the findings	18
Interpretation	#20	Give a cautious overall interpretation considering objectives, limitations, multiplicity of analyses, results from similar studies, and other relevant evidence.	19
None	#nut-20	Report the nutritional relevance of the findings, given the complexity of diet or nutrition as an exposure.	19
Generalisability	#21	Discuss the generalisability (external validity) of the study results	19
**Other Information**			
Funding	#22	Give the source of funding and the role of the funders for the present study and, if applicable, for the original study on which the present article is based	20
Ethics	#nut-22.1	Describe the procedure for consent and study approval from ethics committee(s).	20
Data statement	#nut-22.2	Provide data collection tools and data as online material or explain how they can be accessed	20

None The STROBE-nut checklist is distributed under the terms of the Creative Commons Attribution License CC-BY. This checklist can be completed online using https://www.goodreports.org/, a tool made by the EQUATOR Network in collaboration with Penelope.ai
